# Supplementary material for: SV2A is expressed in synapse subpopulations in mouse and human brain: implications for PET radiotracer studies
Source: Wellcome Open Res. 2025 Aug 11;10:415. [Version 1] doi: 10.12688/wellcomeopenres.24668.1 (PMC12603525; doi:10.12688/wellcomeopenres.24668.1)
Supplement: Peer review history

# Reviewer report: Reviewer response for version 1

**DOI:** 10.21956/wellcomeopenres.27183.r136612

**Published:** 2025-11-10

**Authors:** Bong-Kiun Kaang

**Affiliations:** 1 Institute for Basic Science (IBS), Daejeon, South Korea

The authors investigated whether SV2A, the target molecule of SV2A-based PET that is widely used as a means to detect brain disorders by identifying synaptic pathology, is indeed ubiquitously expressed across synapses. They employed single synapse synaptome mapping based on a well-designed immunohistochemistry to brain tissues from both mice and humans to analyze SV2A expression at the synaptic level. Their results show that SV2A is not expressed in all synapses; rather, its expression varies between excitatory and inhibitory synapses, as well as across different brain regions. These findings strongly suggest that the diversity of synaptic SV2A expression should be carefully considered when interpreting clinical data obtained from SV2A PET imaging.

 The experimental design and data interpretation are sound, and the manuscript is well written. I only have minor suggestions to improve the manuscript:

1. The functional role of SV2A and the rationale or historical reason for its use as a PET synaptic marker would be further described in the Introduction for general readers.
2. I wonder whether the authors analyzed neuronal or nuclear RNA-seq data published elsewhere to compare the expression levels of SV2A and other excitatory and inhibitory synaptic markers at the single-cell transcriptomic level. If so, they may discuss it.
3. Minor copy-editing is needed (e.g., label-ling, mat-ter, find-ings, etc.).

Is the work clearly and accurately presented and does it cite the current literature?

Yes

If applicable, is the statistical analysis and its interpretation appropriate?

Yes

Are all the source data underlying the results available to ensure full reproducibility?

Yes

Is the study design appropriate and is the work technically sound?

Yes

Are the conclusions drawn adequately supported by the results?

Yes

Are sufficient details of methods and analysis provided to allow replication by others?

Yes

Reviewer Expertise:

Synapse biology

I confirm that I have read this submission and believe that I have an appropriate level of expertise to confirm that it is of an acceptable scientific standard.

# Reviewer report: Reviewer response for version 1

**DOI:** 10.21956/wellcomeopenres.27183.r132470

**Published:** 2025-10-06

**Authors:** Sridhar Natesan

**Affiliations:** 1 King's College London, London, England, UK

The authors have employed high-resolution synaptome mapping in adult mouse and human brain tissue sections capturing the expression of SV2A protein. SV2A is protein known for its expression in synaptic vesicles and of lately has been labelled as a surrogate marker for all synapses especially in context of brain PET imaging using radiotracers that selectively bind to it.

 The result of this study shows that SV2A is not present in all synapses but is found in specific subsets and its expression varies across different brain regions. This finding is very important as interpretation of SV2A PET imaging studies in human clinical cases have assumed that SV2A is expressed in all synapses and in a homogenous manner.

 The authors have validated their methodology in an adequate manner especially to determine optimal immunofluorescence labelling conditions of target proteins in the mouse brain. They have also taken adequate steps in the case of human brain tissues.  

 In the mouse brain they have carefully chosen regions with high levels of synaptic diversity and have measured SV2A density along with well-characterized presynaptic/postsynaptic proteins. Their results indicate that SV2A is expressed in subpopulations of excitatory and inhibitory synapses across the mouse brain. This has been extended to the human brain sections with similar findings.

 I have a few observations to make:

  

1. In terms of methodology fluorescence is the key measure in this study. In the case of SV2A in mice brain sections, the secondary Ab is Anti rabbit and they are compared to fluorescence generated from Anti-mouse/guinea pig albeit all generated from Goats. In this context how rigorous is the methodology to compare SV2A puncta density to summed density of PSD95 and GPHN to determine fractional expression based on fluorescence read outs.
2. It is interesting to note that co-localization of SV2A with postsynaptic proteins markers in a smaller proportion also means that it is not uniquely presynaptic as commonly mentioned in the literature.
3. In PET most studies for SV2A compare large ROIs and though it is not right for authors to have claimed “ubiquitously and homogeneously located in synapses across the brain”, findings from PET studies could still hold but need to be carefully interpreted. More research on this is needed to qualify its relationship to pathology.
4. There was no disease models of synaptic loss included in the study and that could have been more interesting.
5. The human brains were from an elderly group but nevertheless helped in understanding SV2A expression in human brains.

Overall, it is well written and adequately referenced.

Is the work clearly and accurately presented and does it cite the current literature?

Yes

If applicable, is the statistical analysis and its interpretation appropriate?

Yes

Are all the source data underlying the results available to ensure full reproducibility?

Yes

Is the study design appropriate and is the work technically sound?

Yes

Are the conclusions drawn adequately supported by the results?

Yes

Are sufficient details of methods and analysis provided to allow replication by others?

Yes

Reviewer Expertise:

Neuroimaging and Psychopharmacology

I confirm that I have read this submission and believe that I have an appropriate level of expertise to confirm that it is of an acceptable scientific standard.
